# Humans and the core partition: An agent-based modeling experiment

**DOI:** 10.1371/journal.pone.0273961

**Published:** 2022-09-01

**Authors:** Andrew J. Collins, Sheida Etemadidavan

**Affiliations:** Department of Engineering Management and Systems Engineering, Batten College of Engineering and Technology, Old Dominion University, Norfolk, Virginia, United States of America; University of Zaragoza, SPAIN

## Abstract

Although strategic coalition formation is traditionally modeled using cooperative game theory, behavioral game theorists have repeatedly shown that outcomes predicted by game theory are different from those generated by actual human behavior. To further explore these differences, in a cooperative game theory context, we experiment to compare the outcomes resulting from human participants’ behavior to those generated by a cooperative game theory solution mechanism called the core partition. Our experiment uses an interactive simulation of a glove game, a particular type of cooperative game, to collect the participant’s decision choices and their resultant outcomes. Two different glove games are considered, and the outputs from 62 trial games are analyzed. The experiment’s outcomes show that core coalitions, which are coalitions in a core partition, are found in about 42% of games. Though this number may seem low, a trial’s outcome is more complex than whether the human player finds a core coalition or not. Finding the core coalition depends on factors such as the other possible feasible solutions and the payoffs available from these solutions. These factors, and the complexity they generate, are discussed in the paper.

## Introduction

Cooperative game theory is a type of game theory that considers games in coalition form [1]. Cooperative game theory is the dominant technique used to model strategic coalition formation. Thus, if a modeler is developing a simulation model of a real-world scenario that contains strategic coalition formation elements, there might be a desire to enrich their simulation model with cooperative game theory concepts [2]. However, it would be prudent to, first, understand the limitations of any cooperative game theory concept used to enrich a simulation model. In this paper, some of these limitations are investigated.

In our research, we consider the cooperative game theory solution concept called the core partition [3, 4], which is concerned with stability. In this paper, we outline an investigation into the difference between the core partition solutions and the final coalitions formed from actual human decision-making. To this end, we collect data using an interactive hybrid simulation of a cooperative game, which involves a single human participant and several computerized agents. The resultant formed coalitions from the experiment are compared to the core partition solutions, and discussion is given on why differences might occur. This research hopefully helps future researchers who are considering using cooperative game theory concepts in their modeling of strategic coalition formation by humans.

Behavioral game theorists have repeatedly shown, through human subject experiments [5–7], the outcomes predicted by non-cooperative game theory are not necessarily descriptive of those generated by actual human behavior. Cooperative game theory is different from non-cooperative game theory, so these results involving non-cooperative game theory do not necessarily translate to cooperative game theory. If cooperative game theory concepts are going to be used in the simulation modeling of human systems, then there is a desire to understand how its outcomes differ from those generated by humans.

Behavioral game theory has focused on two or three players’ normal-form games because of the additional difficulty of creating human subject experiments involving four or more players [8]. Our research considers games of seven players, as a non-trivial cooperative game that requires at least four players. This paper uses an interactive simulation experiment, so only a single human participant is required for each trial, with the remaining players of the games being controlled by computerized agents. The game considered in our research is called the glove game, also known as the shoe game. The glove game is a simple market game that has been used, in the context of cooperative game theory, for human experiments [9, 10] and to exemplify phenomena of interest [11, 12].

Our simulation experiment with cooperative game theory involves 62 interactive simulation trial games. The outcomes of interest are the resultant coalition structures, that is, a partition (into coalitions) of the players for a given glove game. Each trial of our experiment involves a single human participant playing a sequence of different glove games. Since cooperative game theory involves multiple players, the behavior of the remaining players is controlled by computerized agents that are governed by a heuristic algorithm, which was designed to find the core partitions in agent-based simulations [2, 13]. The human participants interact with the computerized agents through a specially designed Graphical User Interface (GUI). Though simple, by cooperative game theory standards, the glove game requires our human participants to go through an extensive training period. After that training, the 31 humans participate in two different sets of trial games where each trial has only one human participant.

The human demographic characteristics impact on the game outcomes (which were shown not to matter) and the decision consistency between human participants and computerized agents (which was shown to be high between them) of this experiment have already been discussed in other papers [14–16]. The focus of this paper is purely on the relationship between the outcomes of the two sets of trial games (human decision outcomes) and the core partitions, which have not been previously discussed in the other papers, and all results here are original.

The novelty of this research is threefold. Firstly, an interactive simulation with computerized agents has not been used before to investigate human behavior in hedonic games (the form of cooperative game theory used in this study). Previous experimentation has exclusively used human participants to play the role of the players; however, since our focus is on trying to understand how strategic group formation can be integrated into a simulation, the use of an interactive simulation is more appropriate in our case. Secondly, our cooperative games are of seven players, unlike older experiments using the glove game that focused on games of three to six players [9]. This increased number of players brings a higher level of complexity to the game. As such, unity measures are unable to capture this complexity; thus, our third novel aspect is that we focus our analysis on studying the coalition structures as opposed to approximate measures of them.

The next section provides a brief background to cooperative game theory, especially the glove game. The agent-based modeling approach is also discussed. The methodology section outlines the trial protocol and model. Finally, the results are presented and discussed, with conclusions given.

## Background

The focus of this background section is to introduce the relevant cooperative game theory concepts. This includes an introduction to hedonic games, which is a form of cooperative game theory. Agent-based modeling and simulation (ABMS) is also introduced, including the ABMSCORE model, which is used in this research to generate the computerized agents’ behavior in the interactive simulation.

### Cooperative game theory

Non-cooperative game theory is, by far, the dominant approach of game theory and uses concepts like the Nash Equilibrium as its solution mechanism [17, 18]. However, the Nash Equilibrium becomes unwieldy when there are three or more players involved. Cooperative game theory was developed to overcome these issues and popularized by Nobel-winning economist Lloyd Shapley. Cooperative game theory is focused on coalition formation and how the payoffs are distributed amongst the coalition members. Its solution mechanisms reflect this purpose, with the main approaches being the Core [19], Shapley Value [20], and the Nucleolus [21].

A coalition is a group of players that have joined together for some purpose. The result of acting on this purpose is represented by a payoff vector. Different forms of cooperative game theory are derived by using different attributes of the payoff vector. For example, if a player is able to transfer some of their payoffs (utility) to another player in their coalition, then the game being played has transferrable utility; otherwise, it is a non-transferrable utility (NTU) which means that players are not able to transfer the payoff they receive to other players [22]. Hedonic games are a form of NTU game where the player’s payoff is solely determined by which other players are in their coalition (which is commonly represented by a preference function over all coalitions [22]). In this paper, we use hedonic games and the solution mechanisms considered are the core partition [3, 4] and Nucleolus.

Classic usages of cooperative games include (1) determining the power dynamics of a voting system, (2) modeling market economies (especially exchange economies), and (3) matching roommates of university students based on their individual preferences [18, 22, 23]. The glove game which is used in our experiment is a market economy game. Solution mechanisms within cooperative game theory focus on the concept of stability. Generally speaking, stability means a coalition structure where there is no profitable deviation for players to defect from their current coalition. However, stability can be described in several different ways based on players’ preferences over coalitions, and, as a result, there are several different solution mechanisms. The most commonly used solution mechanisms are Core [19] and Shapley Value [20]. Since the Shapley value assumes that a grand coalition will form (that is, the coalition that contains all players), it is not appropriate for the hedonic games considered in this research. As such, the core concept is used. In hedonic games, the core partition concept is equivalent to the core concept in standard cooperative game theory [24]. A core partition is a coalition structure where no subsets of players have an incentive to defect from their current coalitions to form a new coalition [25]. Another solution concept, the Nucleolus [21] is also considered in this paper; it always exists and is a member of the core if the core exists. The nucleolus is based on the idea of maximizing the happiness of the most unhappy coalition [18]. Other solution mechanisms exist like the Kernel, bargaining sets, super core, etc.; however, they are not discussed in this paper; please see Thomas [18] for more details on these other solution methods.

#### Core partition

The core is normally described in terms of coalition payoff value in standard cooperative game theory. A core partition in the hedonic game is equivalent to a member of the core [24], which is defined as a coalition structure that is not blocked by any coalition. For a given coalition structure, CS, a potential coalition, C, for instance blocks a CS if every member of the coalition C would earn a higher payoff if they joined the potential coalition C, compared with the payoffs they would receive under their current coalition in CS. Note that the members of the blocking coalition, C, may be located in different coalitions (subsets) in the coalition structure. Mathematically, this is represented as follows using the preference relation of player ‘i’:

$$C\;{≻}_{i}{CS}_{i},\;{CS}_{i}\in CS\;s.t.\;i\;\in {CS}_{i}$$ (1)

A core partition is a covering set of disjoint coalitions where no subset of players has an incentive to form a new coalition [3, 4]. As with the core, it is not necessarily unique, and there is no guarantee of existence; it has been shown numerically that many hedonic games without core partitions exist [26]. The core partitions for the glove games, considered in our experiment, are determined by hand calculation; the games are purposely created to have non-empty cores.

Core partition is not the only solution mechanism in hedonic games. Others include Nash stability, individual stability [4], and strong Nash stability [27], which are differentiated based on different stability criteria. We only consider the core partitions in this paper [3]; the core partition concept is also known as core stability [4].

#### Hedonic games

Hedonic games are cooperative games where players’ payoff is determined by their preferences over their current coalition. The more a player prefers the coalition, the higher the payoff. The mathematical study of hedonic games began simultaneously and independently by Banerjee et al. [3], and Bogomolnaia and Jackson [4]. Hedonic games are the generalization of matching games [28, 29] like the stable roommate problem and the marriage problem [28]. Instead of looking at which pairs will form, hedonic games consider games of any size. Researchers became interested in hedonic games because they model the grouping preferences of individuals [30] and have been applied to practical problems like organizing robotic swarms [31].

The study of hedonic games is concerned with finding stable coalition structures like the core partition already mentioned. As such, hedonic games are also known as coalition formation games [3]. In hedonic games, the players’ payoff is not dependent on what is occurring in other coalitions; only who is a member of their coalition is important. Some important terms relating to hedonic games are defined here:

- Coalition structure: a collection of disjoint coalitions that covers all the players.
- Core partition: a coalition structure that is core stable.
- Core coalition: a coalition in any core partition, especially with regards to a coalition that has a given player as a member.

Since each experimental trial only involves one human participant in this research, analysis of the results focuses on the final coalition that they join or form; thus, we are concerned with whether their final coalition is a core coalition or not. This focus is due to the lack of control the human participant has over the other coalitions that form in the coalition structure.

#### Modeling assumptions on cooperative game theory

As with all traditional game theory concepts, cooperative game theory assumes that its players are Homo Economicus [32]; that is, the player is infinitely intelligent and is completely rational. Humans are neither of these things, and, as a result, we might expect outcomes of plays by humans not to conform to cooperative game theory solution mechanism’s outcomes. As the results will indicate, this is only partially true.

#### Human subject experiments and the core

Nowadays, the use of experiments within a game theory context is collectively known as behavioral game theory. The focus of behavioral game theory is non-cooperative game theory, as opposed to cooperative game theory; we believe that this focus is due to most non-cooperative games only requiring two players, which makes experiments easier to conduct with the multiple trials required for statistically significant results. However, experiments that do consider cooperative game theory exist.

The main focus of experiments using cooperative game theory is to understand human behavior better, e.g., Bolton and Brosig-Koch [33] tried to understand what coalitions form. There are very few papers that have conducted these experiments for other reasons; for example, Berl, McKelvey [34] conducted an experiment on an NTU cooperative game to determine the validity of the core with regards to human play. Most experiments focused on understanding how certain phenomena affected human decision-making with regards to coalition formation, i.e., power and communication. Studies like Neslin and Greenhalgh [35] and Montero, Sefton [36] looked at the effects of power. Communication and information sharing were considered by Murnighan and Roth [10], Bolton, Chatterjee [37], and Beimborn [38].

There are two main differences between these examples of the use of cooperative game theory in human experiments and our application. Firstly, none of the examples use an interactive simulation approach. This means that our experiment only uses one human participant per trial, and the other players are computerized agents controlled by a specific algorithm which will be discussed later. A brief description of the computerized agent is that they will join suggested coalitions if it provides an increase in utility for them, and they make coalition suggestions by randomly picking a coalition from all possible coalitions that could form (of a particular type determined by the algorithm).

Secondly, because we use computerized agents, we are able to consider a larger number of players. The majority of papers discussed above consider only three players, the minimum needed to be considered in cooperative game theory. The trial games considered in this paper have seven players.

A similarity between our experiment and the others described is the use of the glove game, which has been used in some classic experiments [9, 10]. However, the use of the glove game is very different in our application whilst Murnighan and Roth [9] used it to gain an understanding of the effect of communication ability and group, we are using it to determine whether human play results in a core coalition being reached.

### Glove game

The glove game is a simple market economy game where the only commodity is gloves, both left-handed and right-handed gloves [12]; all the left-handed gloves are identical and similar to the right-handed gloves. Each player is endowed with a certain number of each type of glove, and they are able to sell pairs for a profit. It is assumed that a coalition of players is able to pool their resources and split the profit. For obvious reasons, it is also known as the shoe game [9, 10]. The version of the glove game that we use in our experiment has two particular features: it is assumed that the profit is shared evenly amongst the members of the coalition no matter how many gloves they bring to the coalition’s pool (this assumption makes the game NTU because players are unable to transfer their payoff to other members of their coalition), and the leather trader (who can sell individual gloves for leather and does not need pairs) is not considered. As such, the payoff for player ‘a’ in coalition ‘S’ is represented by:

Ua,S=min∑b∈SLb,∑b∈SRbS (2)

Where L(.) is the number of left-handed gloves of a player, and R(.) is the number of right-handed gloves.

By cooperative game theory standards, the glove game is quite simple; however, it can be quite difficult for novices to understand, as we discovered in a prototype of our experiment [39]. Due to our experiment trial process, participants are given extensive training in the mechanics of the glove game. We provide a simple example here for the reader’s benefit.

Consider a game with glove traders: Abbie, Billy, and Charles. Abbie has two left-handed gloves and zero right-handed gloves, represented by <2,0>. Billy has <0,3> and Charles has <2,2>. If Abbie and Billy form a coalition, AB, then they will have two pairs and gain a payoff of one each. If all players form a coalition, ABC, known as the grand coalition, they will have four pairs and a payoff of 1.333 each. If Charles forms a singleton coalition, C, then he will gain a payoff of two. As such, Charles does not want to join the grand coalition, so he would rather remain in his singleton coalition. Abbie and Billy will get zero payoffs if they remain on their own, so they would rather form a coalition. As a result of this, a stable coalition structure of this game is (AB)(C), with Abbie and Billy getting a payoff of one and Charles a payoff of two. A stable coalition structure, in this context, is a core partition.

The human participants in our experimental trials playa total of seven different glove games, ranging from three players to seven players. The first five are training games similar to the simple glove game described above. The final two games are of seven players each. It is the results from these final two games that are used in the analysis presented in this paper. One of these two final games had only a single core partition solution, whereas the other had multiple core partitions.

#### Glove game with the single-core partition

In this paper, the first game we will consider for analysis is a game of seven players that only has one core partition. The allocation of gloves is shown in Table 1; for example, player ‘E’ has three left-handed gloves and two right-handed gloves.

**Table 1:** Allocation of gloves to the seven players in the game with a single core partition.

	A (Human Player)	B	C	D	E	F	G
**Left-handed Gloves**	2	1	4	1	3	0	2
**Right-handed Gloves**	1	2	0	3	2	3	3

As we mentioned before, our concern is the final coalition of the human player (player ‘A’). Using the coalition notation of Bonifaco, Inarra [40], where, for example, ABG refers to the coalition of players ‘A,’ ‘B,’ and ‘G’ only; we can construct a preference table for player ‘A’ as shown in Table 2. A player prefers one coalition to another if they get a higher payoff from it. Hedonic games are games that use this type of preference [3, 4].

**Table 2:** Coalition preferences of the human player (A) in the game with a single core partition.

Coalition	Reward per member
ACDG	1.75
AG, ACE, ADE, AEG, ABEG, ACDE, ADEG, ACDEFG	2
ABCDEFG	1.857
ABCEFG, ABCDEF	1.833
ABCDG, ABCFG, ABDEG, ACDEF, ACDEG, ACEFG	1.8
ACDF*, ABDE, AEFG	1.75
ABE, ABG, ADG, AEF, ABCDFG	1.667
ABCDE, ABCDF, ABCEF, ABCEG, ABEFG, ADEFG	1.6
AB, AD, ABCD, ABCF, ABCG, ABDG, ABEF, ADEF, ABDEFG	1.5
ABDEF	1.4
ABD, ACD, ACG, AFG	1.333
ABCE	1.25
ABDFG	1.2
A	1

Table 2 shows all the coalitions that are an improvement on player ‘A’ remaining in a singleton coalition (which has a reward of one). To determine player ‘A’s core coalition, we must first determine the core partition of the game. This can be achieved through an exhaustive search (i.e., comparing every coalition to every coalition structure to see if it is blocked). We next discuss the properties of the game so that the reader can better understand the derivation of the core partition.

An astute reader will notice from Table 1 that coalition EG will block any coalition structure not containing it because these players earn a reward of 2.5 from EG. As such, the core partition must contain EG. This requirement means that player ‘A’ is not in coalition with either player ‘E’ or ‘G,’ which eliminates 75% of the possibilities based on Table 2. Of the remaining possible coalitions, the coalition ACDF provides the highest payoff for all its members. This leaves player ‘B’ in a singleton group. Hence the core partition is (ACDF)(B)(EG), and the core coalition for the human player is ACDF.

#### Glove game with the multiple core partitions

The previous game example had only one core partition; the following example game has four core partitions. This means four coalition structures are stable. The game is shown in Table 3.

**Table 3:** Allocation of gloves to the seven players in the game with multiple core partitions.

	A (Human Player)	B	C	D	E	F	G
**Left Gloves**	1	4	1	0	2	2	2
**Right Gloves**	3	0	3	4	2	1	0

There are several features that are worth pointing out from this game. The highest payoff that a coalition can achieve is two per player (this is because no player has more than four gloves, which would be required of most coalitions to obtain a payoff greater than two). Thus, any coalition that obtains a payoff of two per member is stable. There are five such coalitions: (ABC), (ABCE), (BD), (BDE), (E). As with the single-core partition game, we construct the coalition preferences for player ‘A,’ shown in Table 4.

**Table 4:** Coalition preferences of the human player (A) in the game with the multiple core partitions.

Coalition	Reward per member
ABC*, ABCE	2
ABCEF, ABDEF, ABDEG	1.8
ABCF, ABDE, ABDF, ABDG	1.75
ABCDEFG	1.714
ABD, ABE, AEF, AEG, ABDEFG, ABCDFG, ABCDEG, ABCDEF	1.667
ABCDE, ABCDF, ABCDG, ABCEG, ACEFG	1.6
AB, AE, AF*, AG*, ABCD, ABDG, ABEF, ACEF, ACEG, ACFG*, AEFG, ABCEFG	1.5
ABF, ACE, ACF, ACG, AFG, ACDEFG	1.333
ABEG, ADEF, ADEG	1.25
ABEFG, ACDEF, ACDEG, ACDFG	1.2
A	1

Before we discuss the core coalitions, there are several properties of the game that require discussion. Firstly, it should be pointed out that games always start with each player assigned to their singleton coalition. Since Player ‘E’ starts with a payoff of two, they will never leave their singleton coalition. As such, any coalition that involves player ‘E’ can be ignored, approximately half of possible coalitions, thus they are greyed out in Table 4.

It should be noted that Player ‘B’ is the lynchpin to the high payoff coalitions. There are two coalitions that Player ‘B’ would like to be a member of (ABC) and (BD) (that did not contain player ‘E’). Since the other players involved in these coalitions would obtain the highest payoff, they also would like to be a member of them. As such, it is simply a matter of which one is proposed first, as once Player ‘B’ is in one of the two, they will not move to the other.

Noting that once players ‘B’ and ‘E’ chose their coalition to find the core partitions simply becomes a case of determining the coalitions that maximize the remaining players’ payoff. This results in core partitions: (ABC)(DFG)(E), (ACFG)(BD)(E), (AF)(BD)(CG)(E), and (AG)(BD)(CF)(E).

The game actually could have eight core partitions because if the two coalitions (BDE) or (ABCE) could be formed it would result in the payoff of two per player. However, since Player ‘E’ in its singleton coalition has gained the payoff of two, there is no incentive for it to join either coalition. So, these two coalitions can be ignored.

### Agent-based modeling and simulation

In this research, during each experimental trial, the human participant interacted with computerized agents using a specially constructed Graphical User Interface (GUI). The computerized agents existed in an agent-based simulation environment with a human-in-the-loop. ABMS was the appropriate modeling paradigm because it allows for autonomous and heterogeneous agents [41].

ABMS is a Modeling and Simulation (M&S) paradigm that uses computer simulation to investigate how micro-level processes can generate macro-level emergent outcomes [42]. Modelers can give instructions to hundreds or thousands of “agents,” all operating independently [43]. ABM has long been advocated as the appropriate approach to modeling complex adaptive systems [44] like a game. Human systems are complex adaptive systems due to human nature, and, as such, ABM has been advocated as a method to model human behavior [42]. ABM has become one of the main techniques used in computational social science. It has been used to model a wide variety of systems, from evacuations [45] to foreclosure contagion in housing markets [46] to crime [47] and armed conflict [48].

One noticeable exception to the widespread use of agent-based modeling within the social sciences is economics. Though it has been repeatedly advocated as an appropriate modeling technique [49, 50], the resistance to its use comes from its inability to model the strategic behavior of multiple interacting agents. Game theory is the dominant technique for modeling strategic interactions of multiple decision-makers, as we have already discussed, and is, thus, widely used in economics. Using the core concept, we develop a hybrid approach that enriches agent-based modeling with strategic behavior. Enrichment is when a modeler "aims to enhance one method (the main method) by using elements of another” [51].

To enrich an agent-based model, Collins and Frydenlund [2] developed an algorithm that allows agents, within a computerized simulation, to make coalition suggestions and to strategically choose whether to join or leave a coalition. This algorithm was improved upon by Vernon-Bido and Collins [13]. It is this improved version of the algorithm that is used in our simulation during the experimental trials. The algorithm is referred to as the ABMSCORE.

The validity of the algorithm has been tested against the ideal coalition structure [13]. It is also shown to be generally consistent with human play [16]. The model section provides more details.

We should note that it is not the first time that researchers have used computerized agents and human experimentation to validate the results of the algorithm that controls computerized agents. For instance, in Roth and Murnighan [52], human subjects play prisoners’ dilemma games against programmed opponents to assess the expected duration of the game. Also, in a buyer-supplier environment, human subjects took the role of sellers while the buyers were controlled by computerized agents to check contestable market theories [53, 54]. These examples differ from our research because either the role or environment is different between the human player and the computerized agent. Duffy [55] believes that agent-based computational economic researchers should validate computerized agents’ behavior by conducting human subject experiments in the same environments as computerized agents as this paper does.

## Methodology

To determine whether the game solutions provided by cooperative game theory describe the outcomes of the game when played by humans, you could simply collect data from those games played by humans and compare it to the cooperative game theory outcome. As discussed in the”Human subject experiments and the core” section, many have done this. However, there are two key limitations to this approach: firstly, it is costly, both in terms of time and resources, to run trials that involve several human participants; and, secondly, the more participants you have in a trial, the more difficult it is to account for the complex social interactions that occur. This second issue could be overcome by not allowing any social contact between participants (assuming signaling was not allowed in the game).

Our approach to investigating whether human play outcomes can result in cooperative game theory solutions (core partitions) being formed is to construct an interactive simulation of the glove game and conduct an experiment to collect data on the resultant coalitions formed from a human player’s play. Strictly speaking, our approach is not a human subject experiment, and, as a result, our findings provide evidence, not proof of the phenomenon we discuss. The limitations of our approach are discussed in the limitations section of this paper.

Since a cooperative game involves several players, the other players had to be simulated. These simulated players’ decisions were controlled by the ABMSCORE, which was shown, in certain experiments, to converge to the cooperative game theory solutions 96% of the time [13], so we assume that the simulated agents’ play is consistent with cooperative game theory.

The advantage of using simulated agents is that a larger number of players can be considered in the game. Normally, the cooperative game theory experiments only considered three-player games; our focus is on seven-player games though we could have gone much larger. We appeal to the Law of Parsimony for our justification for considering only one human player and seven players in total in a game. As our results provide positive indicators of the use of cooperative game theory, future research could involve a game of much larger player count; however, there is the issue of computational intractability of actually finding the cooperative game theory solution to compare to the results of the future experiment of games with more than 20 players [56].

Our experiment uses the glove game described above as its case study to compare the resultant human-involved coalitions with the theoretical coalitions of cooperative game theory. Each trial involves a single human player who participates in several games. Each game is played over several runs, where the players form new coalitions based on the suggestions they and the other players make during that round. Once the human player is happy with their coalition, the game ends, and the coalition structure, formed by the players, is the experiment’s output.

The recruitment of human participants was through word-of-mouth advertising; that is, advertising postings were sent through the researchers’ existing professional and social networks. An Internal Review Board (IRB) exception was made since no identifiable information was collected from human participants. An exemption review was conducted for this research by Old Dominion Universities (ODU) Institutional Review Board (IRB). The study was deemed exempt from full IRB review due to the use of only benign educational tests, and no personally identifiable information being collected (category #6.3). IRB project number and title are [1551613–3] Comparing Human Behavior to Simulated Agents in Coalition Formation.

We should note that, after humans are recruited, they are required to read the “Informed Consent Document” the purpose of which is to give human participants information that may affect their decision whether to agree to participate in the research and to record the consent of those who say “Yes” verbally. This study did not include minors as the human participants were all above 18-years-old. The participants’ ages ranged from 18 to 60+; and less than half the participants were currently students. As such, the education level spanned from from high school diploma to doctorate. The detailed demographic information of human participants can be found in Collins and Etemadidavan [15]. We should note that we previously concluded that none of the human demographic characteristics that we collected (age, gender, education level, game theory experience, and gaming experience) seemed to impact the resultant coalitions formed by human participants and their related behavior [14, 15].

The remainder of this section is dedicated to providing the details of the experiment. The next section describes the protocol for each trial. This is followed by a discussion on the data analysis details, i.e., hypothesis.

### Experiment protocol

**Fig 1:**
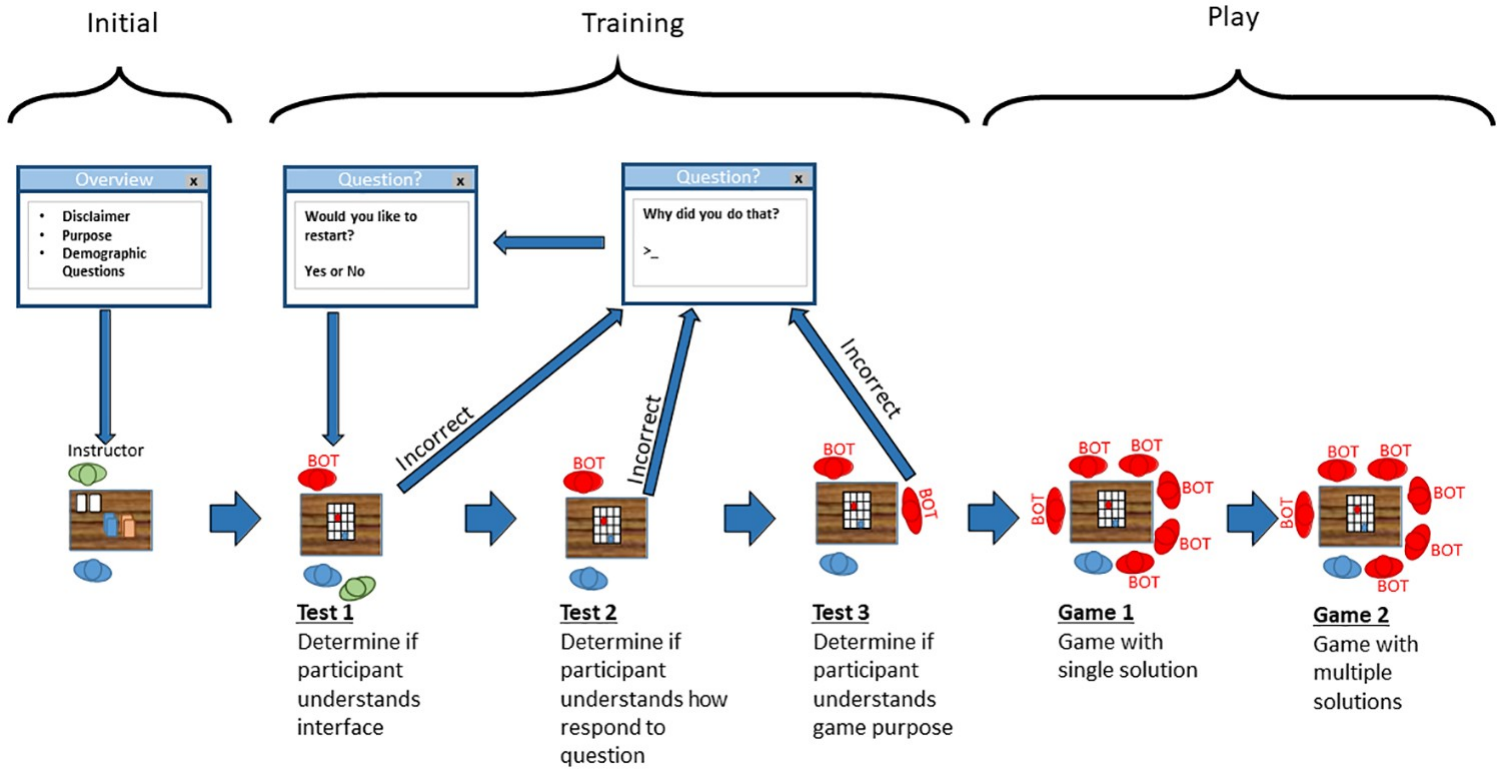
Flow diagram of the experimental trial adapted from Collins and Etemadidavan [14].

The experiment’s human participants are led through three phases: initial, training, and playing (trial). Each phase is divided into several sub-activities, and an overview of the sub-activities is given in Fig 1. The initial phase introduces the participant to the experiment and disclaimer; it then asks the participant a series of demographic questions by a questionnaire and, finally, explains the glove game rules and policies. The training phase requires the participant to play several training games to determine if they understand the computer interface, GUI, and the rules of the game. Finally, the human participant plays the two trial main games. Each phase is described in detail in its relevant section below.

#### Initial phase

The initial phase of a trial is designed to remove all other necessary elements so that the human participant can focus solely on the glove games in the later phases. These elements include meeting and being introduced to the moderator, explaining the premise of the glove game and that the participant has no obligations to undertake the experiment trial and can leave anytime, and asking the participant some demographic questions by a questionnaire. The demographic information of human participants can be found in Collins and Etemadidavan [15].

Initially, the experiment was expected to be developed as a web-based game; however, our initial prototype demonstrated that the glove game was too complex to be explained through a web interface [39]. Though simple, in game-theoretic terms, it was clear that some participants did not understand the mechanics of the game without supervisory support. As such, a decision was taken to introduce a human moderator into the experiment protocol, who would follow a predetermined script. The moderator explains the rules and guides the participant during the training games. They also determine whether the participant is ready and able to proceed to the trial games. The moderator only helps the participant during the trial games if there is a technical problem (e.g., the program crashed). The moderator also helps the participant fill out the demographic questionnaire.

Initially, we thought that different demographic characteristics in human participants would lead to different experiment outcomes. This was shown to not be the case [15], and, as such, we will only briefly discuss it here. For more details on these questions, please refer to Collins and Etemadidavan [15].

Demographic information collected from the participant includes age, gender, education level, and game experience. The game experience is collected to determine how well the participant understands gaming mechanics and, more importantly, how to manipulate them to their advantage. One of our original hypotheses was that those with game theory experience would be more likely to understand the glove game mechanism and how to obtain a higher payoff within the game, but this was shown not to be true [14]. Please see Collins and Etemadidavan [14] for more discussion on this.

The only other information collected from the participants are their plays (decisions and coalition structures that were formed) during the trial games, which are automatically recorded by the simulation.

#### Training phase

During the prototype experiment [39], it was found that some participants had difficulty understanding the glove game mechanics; as such, our experiment protocol includes an extensive training period. During this training period, the participant plays five glove games. Two of these games are verbally presented, by the moderator, during the initial phase. The final three are presented using the GUI interface during the training phase. These final three training games are also used to evaluate whether the participant understands the glove game rules or not and whether they understand the GUI interface. In the training phase, the participants demonstrated proficient knowledge. The training games involve at most three players, and the trial games involve seven.

#### Playing (trial) phase

The final two games the participant plays are the two glove games presented in the background section. These two games are played on the GUI interface, which automatically records, at each round of the game, all decisions and choices as well as the coalition structures which are formed. It is these coalition structures, formed from the play of these two games, that are the basis of the analysis presented in this paper.

All the games, both testing, and trials, are played over several rounds. A game round involves the human player making coalition suggestions followed by the computerized agents making suggestions. If these suggestions are acceptable to all agents in the new suggested coalition, then that coalition forms. A detailed description of a round of the game can be found in the model section below.

All the data collected from the training and trial games played in GUI can be found at https://www.comses.net/codebases/3039b5b4-9a52-4195-a444-0a3a87ef229d/releases/2.5.0/ (accessed on 24 September 2021).

### Data analysis

The focus of this research is on strategic coalition (or group) formation. Thus, the focus of the data collection is on coalitions and coalition structures. Specifically, whether the coalition formed in the experiment’s outcome is either a core partition or core coalition. A core coalition is a coalition that is a member of a core partition.

The outcome of a game is always a coalition structure, a collection of disjoint coalitions that cover all the players. A coalition structure is thus also a partition of the players. A core partition is a coalition structure that satisfies the core criteria (i.e., no subset of players has an incentive to form a new coalition of themselves). If each coalition is given a unique number label, a coalition structure can be represented as a sequence of numbers which represent each player’s coalition in player order. For example, {0, 1, 0} represents the coalition structure where the first and third players are in coalition “0” and the second player is in coalition “1” (which is a singleton coalition). The different coalitions found from our trials are outlined in Tables 5 and 6 in the *“Result”* section. We use the partition number convention outlined in Djokić, Miyakawa [57]; this means that the human player is always in coalition “0.” Using this notation, we are able to analyze the final partitions of the games.

**Table 5:** Final coalition structures for the game with a single core.

	Human Player (A)	B	C	D	E	F	G	Reward	Core Coalition
**Left-handed Gloves**	2	1	4	1	3	0	2	-	
**Right- handed Gloves**	1	2	0	3	2	3	3	-	
**Core Partition**	0	1	0	0	2	0	2	1.75	7
**Non-core**									
**Type 1.5a**	0	0	1	various	various	various	various	1.5	16
**Type 1.5b**	0	1	various	0	various	various	various	1.5	4
**Type 1.5c**	0	0	0	1	2	0	2	1.5	1
**Type 1.33**	0	0	1	0	2	1	2	1.33	1
**Type 1a**	0	1	2	2	3	2	4	1	1
**Type 1b**	0	1	1	1	2	0	2	1	1

**Table 6:** Final coalition structures for the game with multiple core partitions (note that for type 1.75, either player F or G was included in the human players’ coalition).

	Human Player (A)	B	C	D	E	F	G	Reward
**Left Gloves**	1	4	1	0	2	2	2	-
**Right Gloves**	3	0	3	4	2	1	0	-
**Core 0**	0	0	0	1	2	1	1	2
**Core 1**	0	1	0	1	2	0	0	1.5
**Core 2**	0	1	2	1	3	2	0	1.5
**Core 3**	0	1	2	1	3	0	2	1.5
**Non-core**								
**Type 1.75**	0	0	1	0	2	0	0	1.75
	
**Type 1.66**	0	0	1	0	2	3	1	1.66
**Type 1.5**	0	0	1	2	3	4	5	1.5
**Type 1**	0	1	2	1	3	4	5	1

Our analysis focuses on simple descriptive statistics. The underlying assumption of most statistical tests meant that they were not appropriate for all game situations. Due to the exceptional non-linear nature of our game, which is true for most non-trivial games of interest, analysis was done by directly analyzing the results cases. Fortunately, the number of cases considered is low, making our direct discussion approach feasible.

By conducting some statistical tests, comparing the performance of the two games using McNemar’s test [58], determines whether there is consistency in human play across the two games. This test allowed us to determine if there was consistency in human play across the two games. The outcome of this test can be seen in the “*Results*” section.

### Model

The agent-based model implements the glove games (discussed in the “Background” section) into a computerized environment which allows the human player, through a Graphical User Interface (GUI), to play the glove game against computerized agents. This section provides an overview of the model and its flow.

#### Summary of game flow

First, we give a brief overview of the game as it will be played. It is hoped that this overview will give the reader context before discussing our results.

There are two types of choices that a player, human or computerized agents, makes throughout the game. The first type of choice is the coalitions that they suggest to other players. The second type of choice is whether the player chooses to join or not join a coalition suggested by other players. Thus, throughout the game, the players will make a series of choices.

Initially, all players in the game are in their singleton coalitions. The human player (or computerized agent) gets to suggest a coalition involving the other players. If all the other players in the suggested coalition accept this coalition (because it increases their utility), then the suggested coalition forms. If any of the players in the suggested coalition reject it, then the coalition does not form. Note that only the players in the suggested coalition are asked if the coalition should form; no consideration is given to the other players even if the suggestion breaks up their existing coalition.

After the human player’s turn, the other players (computerized agents) get an opportunity to suggest a coalition based on rules which we will describe later. Suppose all the players in the suggested coalition agree, then the new coalition forms. Once the other players have made their suggestions, and the resultant coalitions are formed, then a new round begins with the human player making coalition suggestions followed by the other players’ suggestions. This alternation of suggestions continues until the human player is satisfied with the game state.

#### Human player suggestions

The human player makes a coalition suggestion by selecting the players they wish to form a coalition. This can include members of their current coalition, and it is assumed to include themselves. Any combination of players is acceptable. Once a coalition is selected, the computerized agents involved determine if their utility (payoff) would increase under the new coalition. If all computerized agents’ utility (payoff) would increase, then the new coalition forms; otherwise, the coalition suggestion is rejected. The human participant can also decide to stay in its singleton coalition (or current coalition) without making a coalition suggestion to others. GUI is the platform to handle these decisions.

**Fig 2:**
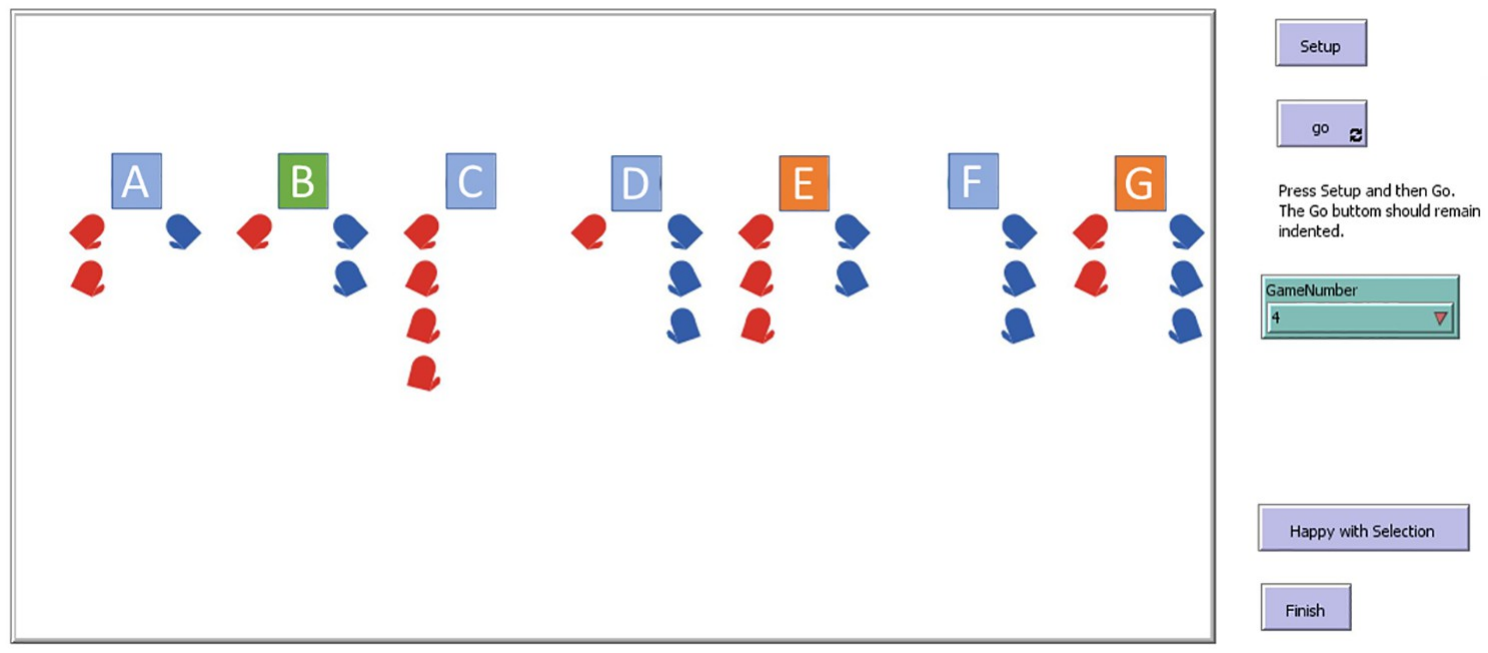
Graphical User Interface (GUI) used in the simulation. The agents are represented by alphabetic squares, while the human player is always player ‘A’. The colors of the squares represent the current coalition of the agents. For instance, players ‘E’ and ‘G’ are in the same coalition, and the payoff for each of them is 2.5. The left-hand gloves are shown in red and the right-hand gloves in blue. For instance, player ‘A’ above, has two left-hand gloves and one right-hand glove.

The purpose of the GUI is to give insight to the human player about the game environment and let them suggest coalitions or respond to suggested coalitions. Additionally, it allows them to see the number and type of gloves (left-hand or right-hand) that other computerized agents have to suggest or respond to a coalition. The graphical representation that the human player sees is shown in Fig 2.

To suggest coalitions the human player clicks on desired computerized agents’ squares to propose a coalition and then selects the “happy with selection” button. If all computerized agents agree/one disagrees to form that coalition then the coalition would form/not form and the next round begins with the coalition suggestions of the computerized agents. The human player can play the game for as many rounds as they desire and when they decide to finish the game, they simply press the "Finish" button to end the game.

#### Computerized agent suggestions

After the human player’s suggestion is resolved, the computerized agents get to suggest coalitions. These suggestions are created using the ABMSCORE described in detail in [13]. Once a suggestion is made, all the computerized agents in the new coalition determine if they wish to join the coalition. If they do and the human player’s agent is not part of the new coalition, then the coalition forms. If they do and the human player’s agent is part of the suggested coalition, then the human player is asked whether they wish to join the new coalition. If the human player does decide to join the new coalition, then it forms. The computerized agents, collectively, make six different coalition suggestions per round. Note that the computerized agents’ suggestion does not have to involve the human player.

Once both suggestion phases are completed, a new round begins, starting with the human player suggesting a new coalition. We should note that the human player can choose to stay in their current coalition and not suggest any new coalition. Using the interactive simulation, we are able to collect the data from the trial games.

All the interactive simulation decisions use the latest version of the ODD protocol [59], and more detail of it can be found in [14].

## Results

A total of 62 trial games were conducted from 31 human participants, and the resultant output data from each trials’ games is discussed in this section. The results are the coalition structures that formed and whether, or not, those coalition structures are part of the core. The results are different for the two games, with only seven trials resulting in finding a core coalition in the single-core partition game (22%), whereas 19 found it in the multiple-core partitions game (61%). It could be argued that the chance of finding a core coalition is higher in a game when more cores are available (as only one needs to be found); however, as the discussion will show, we do not believe it is that simple. As mentioned above, we avoid a hypothesis test approach and focus on a descriptive analysis of why human players do not reach the core coalition. Our discussion focuses on why the human players choose to suffice with non-core coalitions.

We previously concluded that none of the human demographic characteristics that we collected seem to impact the resultant coalitions formed by human participants and their related behavior [14, 15]. As such, the following discussion considers the trial population in its entirety. The two solution mechanisms considered are the core partition [3, 4] and the nucleolus [21]. For the single-core partition game, the core is the nucleolus solution [18]. The nucleolus [21] always exists and is a member of the core if the core exists. The nucleolus is based on the idea of maximizing the happiness of the most unhappy coalition [18].

Not all trials end in a core partition being found. Also, not all trials end in the player being in the correct coalition for a given core partition; however, there could be many reasons for this discrepancy. One reason could be a lack of understanding, by the human players, of the game mechanics; however, as the results below show, we do not believe this is the case for any of the trials (assuming that human players did not stumble upon reasonable solutions by chance). In many cases, we believe that, the players spotted a feasible solution, which they stuck to, and they did not think through its consequence. The remainder of this section is dedicated to the descriptive analysis of these outcomes. This is followed by a statistical comparison of the two trial games.

### Descriptive analysis

The final coalitions and coalition structures found from the trials are discussed in this section. The game with the single-core partition is discussed first, followed by the multiple-core game.

#### Game with the single-core partition

The game with the single-core only has one core partition solution and 31 trial game outputs collected for this game. The results for this game are shown in Table 5. The table first shows the initial endowment of the players, as a reminder, though the discussion is given in the “Glove game” section. The third row and beyond show the different coalition structures obtained from the experiment. These rows show which coalition each player belongs to, the reward (payoff) obtained by the human player, and the number of trials that resulted in that coalition structure. The coalitions are numbered, starting with zero, using the approach outlined in Djokić, Miyakawa [57]. The human player’s coalition is always labeled zero.

Since our focus is on the core coalition of the human player, we are only really concerned with coalitions that contain the human player (coalition zero). As such, we amalgamate the results that have the same coalition for the human player; when there are multiple coalition possibilities for the other players, based on the results, we call those players’ coalitions “various.”

As the table shows, only seven players’ final coalitions were the core coalition (with only four forming the core partition). In contrast, the majority of human players found the AB (Type 1.5a) coalition, which is a reasonable coalition. By reasonable coalition, we mean a coalition that increases a player’s payoff from their starting value. As Table 5 shows, the coalition AB (Type 1.5a) increased the human player’s reward from 1 to 1.5. Forming a coalition with player B fits well for several reasons. Firstly, player B’s endowment mirrors the human player’s endowment; that is, the human player has two left gloves, and player B has two right gloves, and vice versa. Secondly, the pool of gloves generated by AB (Type 1.5a) has an exact number of pairs with no left-over spares, so no wasted gloves. Finally, player B is the next player in the line of players in the GUI, after player A, so it might be the first player that the human player considers for a coalition. We believe that for these reasons, the human player was satisfied with the coalition AB (Type 1.5a) and, as such, did not bother to look for the more complex core coalition (which involves four players). For some of these reasons, one could argue for a efficient fit with player D. As such, the human player was sufficing by choosing player B (or D) as their coalition partner, which accounted for 65% of all final coalitions.

There are four types of observed coalition partitions that were different from those discussed above: *1*.*5c*, *1*.*33*, *1a*, and *1b*. It is not clear why the players formed coalition *types 1*.*5c*, or *1*.*33*, but both types represent an improvement from their initial singleton coalition. For *types 1a* and *1b*, there are different reasons why they settled for a payoff that was the same as their initial endowment. However, we studied both trials and noticed some interesting behavior. In the case of *type 1a*, they had formed a coalition with Player *D* earlier on (to obtain a payoff of 1.5), but Player *D* left them for coalition CDF; now, if the human player had tried to join that new coalition, they would have been accepted and joined a core partition, but instead, they gave up in the next round. In the case of *type 1b*, the human player repeatedly requested that player *E* or *G* form a coalition with them (which neither player would do as it does not benefit them); after several trials, the human player settled on player *F* instead.

We are not the first to observe non-core partitions from human play. Bolton and Brosig-Koch [33] observed many inefficient two-person final coalitions. The situation with the multiple core partitions game was different.

#### Game with multiple core partitions

Of the 31 trial game outputs for the multiple-core game, there were only a limited number of final partitions reached, as with the single-core game. In the multiple-core game, these were either that the human player reached a core coalition or only one of four other partitions. Table 6 shows the partitions outcome for each of these cases, and Table 7 shows the frequency of occurrence. There are some interesting phenomena observed from these results, which we would like to discuss in this section.

**Table 7:** Frequency of coalition structure types.

	**Value**	**Human player in the core coalition**	**Final partition is in the core**
**Core 0**	2	6	2
**Core 1**	1.5	0	0
**Core 2**	1.5	7	3
**Core 3**	1.5	6	1
		**Human player not in the core coalition**	**Final partition is not in the core**
**Non-core**		12	25
**Type 1.75**	1.75	9	-
**Type 1.66**	1.66	1	-
**Type 1.5**	1.5	1	-
**Type 1**	1	1	-

The results from this game differed considerably from the results of the single-core game. To begin with, the majority of players found a core coalition, whereas only a minority found a core coalition in the single-core game. There are several possible reasons for this occurrence. Firstly, since there is only one core coalition, in the single-core game, there are fewer options available to humans. Secondly, since this single core game is the first trial game, it is possible the human participants were “finding their feet” with these more complex games (i.e., the second game benefits from the carryover effect [60]).

Of the 19 trials that resulted in a core coalition being found, only six were core partitions (hence our focus is on core coalitions). *Core 0* is of particular interest because it has the highest payoff for the human player; that is, it is the most desirable outcome; and it was also the nucleolus [21], which is an alternative solution concept to the core. Note that if the core is non-empty, the nucleolus is part of it and the nucleolus always exists. Since our game is NTU, the nucleolus is determined by a computational calculation we have not included here. As Table 7 implies, about a fifth of the players found the nucleolus.

What is interesting about these trial results is what happens to the other 80% of players who did not find the nucleolus. 13 of them found other core coalitions, which provided a reward of 1.5. Of the remaining 12, 11 of them found a better or equivalent reward in their final coalitions. What these results imply is that though the majority of players did not find the nucleolus, they did find a core or a coalition that provides a good reward, even if unstable. Whether the human players knew that they had found an unstable solution is not clear. We will discuss each of the non-core partitions in turn, including the reasons for their instability and possible reasons for why the human players end up in them.

The most common non-core partition was the *Type 1*.*75*, which requires four players: human player, *B*, *D*, and either *F* or *G*. What is interesting about this coalition is that though it rewards well, it requires more players than the best core solution (*Core 0*). It is unstable because if *B* and *D* form a sub-coalition, they will receive a better reward; however, due to the stochastic nature of the algorithm, the sub-coalition formation is not guaranteed. It was not clear if the human players knew about the instability of their game and chose to ignore it. A quirk of the game is that the human player decides when to end it, so they may have ignored the instability because they knew they had control.

*Type 1*.*66* is very similar to *Type 1*.*75*, except it is only three players, and players *F* and *G* are ignored. It suffers from the same problems of instability as *Type 1*.*75*. If the human player had suggested that *F* or *G* joined their coalition, either player would have done so; this implies that the human player did not realize this potential increase in reward.

We believe that the *Type 1*.*5* coalition structure occurred because the human player was interested in finishing the game quickly. They made one simple suggestion and then finalized the game, which did not allow the computerized agents enough rounds to search the coalition space.

We believe that *Type 1* occurred out of frustration from the human player. Previous to finalizing the game, the human player repeatedly requested that player *E* join their coalition. Player *E* was never going to join, as their best outcome is their starting coalition (i.e., their singleton coalition).

### Hypothesis testing

A statistical hypothesis test is conducted to see if the frequencies of finding the core are the same in both games. As mentioned earlier, we suggest that finding the core partition is more probable for the players in the multiple core partitions game. Having dichotomous data, we are able to use McNemar’s Test [58] to compare the two games to see whether the frequencies of finding the core are similar. The contingency table is shown in Table 8.

**Table 8:** Contingency table of results from trials.

		**Singleton Game**	
		**In Core Coalition**	**o/w**
**Multiple Game**	**In Core Coalition**	2	17
	**o/w**	5	7

Since we have a relatively small size, we are able to conduct an exact test version of McNemar’s test, with the probabilities being calculated using the binomial distribution. Using this approach, we find the related statistics (Odd ratio = 3.4, proportion discord pairs = 0.71), and a p-value of 0.004. This means we can reject the null hypothesis of no difference, which supports our claim that there is a difference in the frequency of finding the core coalition. Conducting post hoc power analysis, we find the power to only be 75%, even for a large effect size; therefore, our test is not conclusive.

## Limitations and discussion

There are several limitations with our experimental approach, and some are discussed in this section. This includes the human participant’s ability to take advantage of the game design and the limitations of our experimental design. A discussion is also given on the types of behavior observed by human participants.

### Game design

The experiment reveals that some human participants are able to take advantage of the game design to some extent. For example, the human players’ ability to end the game relatively quickly limits the number of suggestions by the computerized agents. The game design could be adapted to remove this issue by considering a minimum number of suggestions by the computerized agents before the game is ended by human participants; however, what the minimum number of suggestions should be is not obvious, and it would introduce new game design problems (i.e., forcing human players to play when they think they are finished with the game). Generally, it is part of the game design to discover new strategic choices. We are not the first to make this observation within the experimental game theory. Axelrod [6] ran a competition of the iterative prisoner’s dilemma game; it was initially found that the “winning” strategy was not defected, as predicted by game theory, but a “tit-for-tac” strategy. Axelrod’s findings helped in the development of agent-based modeling [61].

### Limitations in the experimental design

The experimental method used for data collection was not a human subject experiment in the strictest sense. Our experiment does not contain any control group and it did not randomly assign the participants to different conditions; therefore, it cannot be considered quasi-experimental research either [60]. As a consequence of our design, we are unable to make any statements about directionality, and it has limited internal validity.

There could be other concerns about the experimental design; for example, we already mentioned the “carryover effect,” which occurred because the human players always played the single-core game before playing the multiple-core partitions game. This limitation, and others, are discussed in detail in Collins and Etemadidavan [14].

However, one limitation has its advantages. Due to having only a small cohort, of 31, we are able to analyze all of the human players’ results individually and, we believe this has given insight into the problem being considered, i.e., what are the differences between the core and coalition formed from human play. Thus, we would propose that our experimental design be seen more as a case-study design to gain insight into the problem.

From our “case study,” we observe some interesting inductive findings. Our results indicate that most players’ final coalition could be explained as either being a core coalition, as desired, or through behavior not anticipated in the games’ design. These results indicate that (1) there is a need to improve the game design, and (2) the core cannot be expected to be found by every human player, even rational ones. Generally speaking, humans do not always act rationally; as such, our research result is another confirmation of the fact that humans find a sufficient solution rather than an optimal one. What is interesting about this research is that there are different types of human behavior being observed.

Since we did not limit the participant pool within the adult population, it is possible that the variation of the results observed could have been due to demographic differences within the participants. We have previously concluded that the demographics of the participants, (i.e., age, gender, education level, game theory experience, and gaming experience) did not seem to impact behavior [14, 15]. However, given the limited number of participants, it is possible that that other demographic differences, or even a more granulated dissection of the population, could have explain the difference in behavior between the participants; as such, future research could be to reconstruct our experiment, based on the insights provided in this paper, and engage a much larger number of participants. This further experiment could provide further insights into the phenomenon observed and, potentially, reinforce the findings discussed in this paper.

### Types of human behavior

The results from our experiment indicate that there is heterogeneity in human behavior concerning the glove game and strategic coalition formation. Some individuals join stable coalitions, some individuals obtain unstable coalitions with higher payoffs, and some individuals focus on joining with players who do not want to join with them. What is interesting is that this behavior is not consistent across the games; for example, only two players found a core coalition in both games.

The deeper question is what drives human behavior? It is possible that the behavior we observe happened purely by chance (if the players just randomly selected their coalitions). However, we do not believe this to be the case; in fact, we hypothesize that the humans are playing quite intelligently and are able to take advantage of the game mechanics. For example, the slowness and randomness of computerized agents to find an appropriate coalition is taken advantage of by the human players choosing to settle with the Type 1.75 coalition in the game with multiple core partitions.

Though simple games are used in this research, that does not mean solutions are obvious. The human player would need to evaluate all coalitions they could be members of to ensure they had reached the best coalition for them. We did not expect the human participants to do this and were not surprised to see that different outcomes occurred because each human used their own heuristic approach to “solving” the game. What would be interesting for future research is to try and uncover what heuristic approaches are used by humans with regard to strategic coalition formation.

To investigate the observed phenomenon in this research (seemingly irrational human behavior), we conducted a structured interview with ten participants after the experiment to better understand their behavior and their strategies. The structured interview comprised questions about the participants’ strategies in playing the games, their satisfaction with the received final payoff, and the meaning of stable coalition in the cooperative game theory. Nine participants stated that they did not care about being a member of the stable coalition, which was clear because they did not know what a stable coalition was except for one of them. Most participants, seven out of ten, chose “Increase my score” as the driving force behind their strategy to join or leave a coalition, one chose “play more rounds in the game”, one chose “finish the game as quickly as possible”, and only one chose “be part of the stable coalition”. In the end, 9 out of 10 felt satisfied with their final score regardless of the different strategies they chose to play the glove game.

Based on the participants’ strategies in playing the game, we can conclude that humans care about increasing their score regardless of whether they ended up in a stable coalition (core coalition) or not. Although the experiment and structured interview results show that humans are not successful in finding the core coalition, they are satisfied with their final received payoff. We should note that, as we do not collect identifiers for the experiment, we cannot connect structured interview results to the experiment outcome based on the individuals. For more details, the questionnaire used can be found at the website location provided in the data availability section of this paper.

Our experimental approach has several limitations, which limit our findings’ conclusiveness. However, our results indicate that some interesting behavior occurred in the experiment, which leads to several possible future research directions.

## Conclusions

This paper describes an experiment to compare the outcomes of a computerized game involving real human decision-making to those outcomes predicated by cooperative game theory. This experiment intends to determine whether cooperative game theory provides a feasible approximation to the coalitions formed by human decision-making. To this end, a particular type of cooperative game, called the glove game, was used with a single human player playing with computerized agents. The results of the experiment indicate that the rates of the human player finding a core coalition are game-dependent, and these rates are significantly different for the two glove games considered.

The two types of glove games were chosen based on their number of solutions. One of the games had a single core partition solution, and the other had multiple core partitions. About three times as many participants found a core coalition in the multiple-core partitions game than in the single-core partition game (19 to 7); however, this phenomenon could have been due to there being more feasible coalitions (as only one had to be found) and the carryover effect. Not all solutions are alike, and only six human participants found the highest payoff core coalition of the multiple-core game, which was also the game’s nucleolus. The participants’ play was not consistent across the two games, with only two participants finding a core coalition in both games.

It should be noted that just because several players did not find a core coalition does not mean their choices are wrong. In the multiple-core game, many found unstable solutions that provided a higher payoff than most core partitions. These human participants took advantage of the game mechanics and opted for a sufficient solution instead of a core solution. One takeaway from our research is that, when modeling humans, allowing the agents to find sufficing solutions might be more valid than allowing them to search for the “optimal” solution.

As the limitations to this research shows, experiments involving humans, in cooperative game theory context, are complex. The research also suggests that humans act heterogeneously in the context of strategic coalition formation, and, as such, agent-based modeling seems to be an excellent approach to model them. Possible further research will include a deeper exploration into the issues highlighted in this paper.
